# Postnatal Catch-Up Growth After Suspected Fetal Growth Restriction at Term

**DOI:** 10.3389/fendo.2019.00274

**Published:** 2019-06-20

**Authors:** Linda van Wyk, Kim E. Boers, Aleid G. van Wassenaer-Leemhuis, Joris A. M. van der Post, Henk A. Bremer, Friso M. C. Delemarre, Sanne J. Gordijn, Kitty W. M. Bloemenkamp, Frans J. M. E. Roumen, Martina Porath, Jan M. M. van Lith, Ben W. J. Mol, Saskia le Cessie, Sicco A. Scherjon, The DIGITAT study group

**Affiliations:** ^1^Departments of Obstetrics, Leiden University Medical Centre, Leiden, Netherlands; ^2^Department of Obstetrics, University Medical Centre Groningen, Groningen, Netherlands; ^3^Haaglanden Medical Centre, The Hague, Netherlands; ^4^Academic Medical Centre, Amsterdam, Netherlands; ^5^Reinier de Graaf Hospital, Delft, Netherlands; ^6^Elkerliek Hospital, Helmond, Netherlands; ^7^Atrium Medical Centre, Heerlen, Netherlands; ^8^Maxima Medical Centre, Veldhoven, Netherlands; ^9^Department of Obstetrics, Monash University Medical Centre, Clayton, VIC, Australia; ^10^Department of Clinical Epidimiology, Leiden University Medical Centre, Leiden, Netherlands

**Keywords:** intrauterine growth restriction, fetal growth restriction, catch-up growth, Disproportionate Intrauterine Growth Restriction Trial at Term, follow-up

## Abstract

**Objective:** The aim of this study was to study growth patterns of children born after suspected fetal growth restriction (FGR) at term and to compare the effect of induction of labor (IoL) and expectant management (EM), also in relation to neurodevelopmental and behavioral outcome at age 2.

**Methods:** We performed a 2 years' follow-up of growth of children included in the Disproportionate Intrauterine Growth Restriction Trial at Term (DIGITAT) study, a Randomized Controlled Trial (RCT) comparing IoL with EM in pregnancies with suspected FGR at term. We collected data on child growth until the age of 2 years. Standard deviation scores (SDSs) for height and weight were calculated at different ages. We assessed the effects of IoL compared with EM and the effects of a birth weight below or above the 3rd or 10th centile on catch-up growth. Target height SDSs were calculated using the height of both parents.

**Results:** We found a significant increase in SDS in the first 2 years. Children born after EM showed more catch-up growth in the first month [height: mean difference −0.7 (95% CI: 0.2; 1.3)] and weight [mean difference −0.5 (95% CI: 0.3; 0.7)]. Children born with a birth weight below the 3rd and 10th centiles showed more catch-up growth after 1 year [mean difference −0.8 SDS (95% CI: −1.1; −0.5)] and after 2 years [mean difference −0.7 SDS (95% CI: −1.2; −0.2)] as compared to children with a birth weight above the 3rd and 10th centiles. SDS at birth had the strongest effect on adverse neurodevelopmental outcome at 2 years of age.

**Conclusion:** After FGR at term, postnatal catch-up growth is generally present and associated with the degree of FGR. Obstetric management in FGR influences postnatal growth. Longer-term follow-up is therefore needed and should be directed at growth and physical health.

**Clinical Trial Registration:** www.ClinicalTrials.gov, identifier SRCTN10363217.

## Introduction

Catch-up growth is considered to be a process of compensatory accelerated growth after a period of poor growth intrauterine. Many studies have shown that growth-restricted children show catch-up growth in the first years after birth (1–6). Catch-up growth is recognized when a child shows accelerated growth, which is visualized as an upward crossing of its centiles in length or weight growth.

Long-term consequences of fetal growth restriction (FGR) and catch-up growth have been studied extensively in the last two decades. It is now generally accepted that low birth weight as a consequence of growth restriction is a risk factor for cardiovascular disease later in life (7–9). It however remains debatable whether this is caused by the catch-up growth itself or the underlying pathophysiology of FGR, by the (extent of) postnatal catch-up growth, or perhaps by a combination. Studies have shown that FGR intrauterine growth restriction itself is associated with an alteration of genetic programming, independent of Body Mass Index (BMI), related to metabolic and cardiovascular diseases (10). On the contrary, others have shown that it is the postnatal catch-up growth and accelerated weight gain that lead to the increased risk (11).

Wit et al. suggest that catch-up growth can be either *complete* or *incomplete* (12). Catch-up growth is considered to be complete in case a mean final height is reached close to the mean target height based on the height of both parents (12). In case of incomplete catch-up growth, the target height range will not be reached and is associated with increased neurodevelopmental problems when compared to children with complete catch-up growth (13–15).

In the Disproportionate Intrauterine Growth Restriction Trial at Term (DIGITAT) trial (16)—a multicenter randomized controlled trial—the effects of induction of labor (IoL) vs. expectant management (EM) in pregnancies suspected of intrauterine growth restriction at term were compared. Suspected intrauterine growth restriction was defined as fetal abdominal circumference below the 10th percentile, estimated fetal weight below the 10th percentile, flattening of the growth curve in the third trimester (as judged by a clinician), or the presence of all three factors.

Data were also collected of women who refused randomization but gave permission for follow-up. Pregnant women with a singleton fetus in the cephalic position, beyond 36 weeks of gestation, and suspected FGR were counseled for trial participation. Primary fetal and maternal outcomes were comparable. Gestational age at birth was lower in the induction group (mean difference –iff (95% CI: –I%fe to –o%fe as was birth weight (mean difference 130 g, 95% CI: –I%f to –o%f *P* < 0.001) when compared to the expectant monitoring group. Despite this difference in (absolute) weight in favor of the children in the group with expectant monitoring, more babies in the expectant group were severely growth restricted, with a birth weight below the third percentile (31 vs. 13%; difference –if%, 95% CI: –I%fe to –o%fer *P* < 0.001) (16).

At 2 years of age, a neurodevelopmental and behavioral follow-up study was performed and showed that children with low birth weights, especially if the birth weight was below the 3rd centile, had a greater risk [adjusted OR 3.6 (95% CI: 1.5–8.8)] for abnormal scores on the Ages and Stages Questionnaire (ASQ). The prevalence of these abnormalities was directly related to the severity of growth restriction (17).

The objective of the present study was to (1) analyze whether obstetric management (IoL vs. EM) policy influences catch-up growth, (2) assess whether children born in the DIGITAT trial (participants and nonparticipants) exhibit catch-up growth until age 2, and (3) assess long-term behavioral and neurodevelopmental outcomes in relation to catch-up growth.

## Methods

### Participants

**Figure 1:**
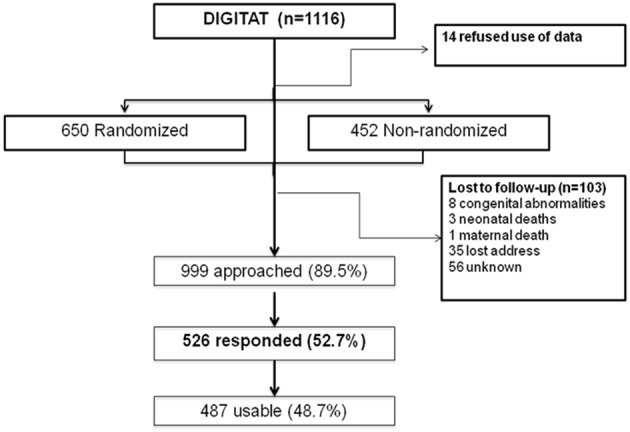
Flowchart of trial participants.

The study population consisted of children born to mothers who participated in the DIGITAT trial (16). During the study period, 1,116 women were eligible for the trial, of whom 14 refused participation, 452 women refused randomization but gave permission for use of their medical data and for follow-up, and 650 women were randomized to either induction of labor (IoL) or an expectant management (EM) (Figure 1). Both the randomized and nonrandomized women are included in this study.

### Baseline Characteristics

Baseline characteristics of the mothers and children were collected at study inclusion and at birth and have been described elsewhere (16).

### Procedure

We collected growth data until the age of 2 of children born to mothers of the DIGITAT trial. General health, height, weight, and developmental milestones of children in the Netherlands are assessed regularly and free of charge in a national program at the Child Health Center's periodically. Parents were asked to participate in a postal inquiry and to send us the recorded growth data (height and weight) of their child until the age of 2 years and to report the height of the father. Maternal height was already recorded at baseline. Standard deviation scores (SDSs) or *Z* scores were calculated for height, weight, and weight for height at different ages based on, respectively, calculated target height- and population-based growth curves (18). Head circumference was not recorded.

Neurodevelopmental and behavioral outcomes of these children were also assessed at 2 years of age using two standardized parental questionnaires: the Ages and Stages Questionnaire (ASQ) (19) and the Child Behavior Checklist (CBCL) (20). The mean scores of both questionnaires were compared between groups, and the numbers of children with abnormal scores were also compared.

We found no commonly used or accepted definition of catch-up growth, and different studies use different definitions. We defined “complete catch-up growth” of height if the measured height SDS minus the target SDS was above −1.6. For weight and for weight for height, an SDS above −2 was used as an indication of complete catch-up growth (21).

### Statistical Analysis

SDSs at birth were calculated using birth weight and height centiles, corrected for gestational age, using the formula of Niklasson (22). “Growth Analyser” v. 3.5 was used to calculate SDSs for height and weight at different ages using Dutch population-based curves (18). The formula of van Dommelen et al. (23) was used to calculate target height SDSs. This formula includes the height of both parents.

Continuous variables were presented as means with standard deviations (SDs), or medians with interquartile ranges (IQRs). Differences between groups were presented as differences in means or percentages with 95% confidence intervals (CIs). Continuous variables were compared using the Student's *t-*test or the nonparametric Mann–Whitney *U* test. The chi-square test and the Fisher's exact test were used for categorical variables.

We assessed whether catch-up growth was influenced by the following factors: IoL compared with EM (only for children included in the randomized study), the effects of birth weight below the 3rd or 10th centile, adverse neonatal outcome at birth (defined as 5-min Apgar score of <7, umbilical artery pH of <7.05, or admission to neonatal intensive care), birth weight centile, maternal smoking, breastfeeding, and gestational age at birth. Parents of the children included in the trial completed two questionnaires, the Ages and Stages Questionnaire (ASQ) and Child Behavior Checklist (CBCL), for presence of neurodevelopmental and behavioral problems, respectively.

The target height SD score was compared to the actual SD score using a paired *t*-test. Changes in SDS scores between different time points were calculated and compared between subgroups using non-paired *t*-tests. Repeated SDS measurements were analyzed using a linear mixed model, which accounts for missing values during follow-up with random person and age effects and fixed group effects. Since the relation between age and SDS scores was expected to be nonlinear, we used quadratic spline functions with knots on 1, 3, 6, 9, 12, and 18 months to model this relation. The results of these analyses are presented graphically.

We performed a logistic regression analysis to study the effect of gestational age and birth weight SDS. In a second analysis, we studied the additional effect of catch-up growth on the outcome. Multiple imputation was used to handle missing observations.

Analyses were carried out in Statistical Package for the Social Sciences (SPSS) 20. The repeated-measures analyses were carried out in R, version 3.02.

## Results

Baseline characteristics of respondents (completion of questionnaires) and non-respondents are shown in Table 1. Respondents were slightly older, smoked less, higher educated, and more often Caucasian than non-responders. Baseline characteristics of the IoL group vs. EM are shown in Table 2. Babies born after EM were more severely growth restricted (birth with below the 3rd centile) than babies born after a policy of IoL (65.2 vs. 75.7%, *p* < 0.05).

**Table 1:** Baseline characteristics.

**Characteristic**	**Respondent (*n* = 526)**	**Non-respondent (*n* = 473)**	**Difference in % or mean (95% CI)**
Maternal age (years)	29.7 (26.3; 33.8)	27.7 (23.3; 31.9)	2.2 (1.5; 2.8)^**^
Maternal Body Mass Index at study entry^†^	21.8 (19.7; 24.6)	21.8 (19.5; 24.8)	0.04 (−0.6; 0.7)
Maternal smoking in pregnancy^§^	134 (25.5)	201 (44.7)	−17.1 (−23.2; −11.1)^**^
Caucasian^‡^	448 (91.1)	354 (73.9)	17.2 (12.5; 21.8)^**^
**EDUCATION**			
Lower professional	200 (69.9)	243 (81.8)	−11.9 (−18.8;−5.0)^**^
Higher professional	86 (30.1)	54 (18.2)	11.9 (5.0; 18.8)^**^
Gestational age at birth	272.3 (265.6; 280.4)	272.5 (264.6; 280.8)	−0.02 (−1.2; 1.2)
Male sex	186 (38.9)	198 (38.4)	−0.4 (−5.673, 6.454)
Birth weight (grams)	2,505 (2,215; 2,771)	2,505 (2,281; 2,769)	30.0 (−77.4; 19.2)
Birth weight <10th centile	382 (72.7)	336 (71.1)	1.5 (−4.0; 7.2)
Birth weight <3rd centile	121 (25.4)	129 (25.2)	0.2 (−5.2; 5.7)
Composite adverse neonatal outcome	30 (5.7)	24 (5.1)	0.6 (−2.2; 3.4)

** *p <0.001*.

*CI, confidence interval*.

*Table shows median [IQR: 25th to 75th percentile) or number (%)]*.

*Data were compared using the Student's t-test, chi-square, or Fisher's exact test*.

† *n = 457 for respondents; n = 394 for non-respondents*.

§ *n = 485 for respondents; n = 449 for non-respondents*.

‡ *n = 492 for respondents; n = 479 for non-respondents*.

**Table 2:** Baseline characteristics of randomized participants (induction vs. expectant monitoring).

**Characteristic**	**Induction (*n* = 158)**	**Expectant monitoring (*n* = 134)**	**Difference in % or mean (95% CI)**
Maternal age (years)	28.03 (25.13; 32.07)	28.19 (24.69; 31.51)	−0.14 (−1.28; 0.99)
Maternal BMI at study entry^†^	22.1 (19.9; 25.8)	22.3 (20.2; 25.3)	−0.05 (−1.26; 1.15)
Maternal smoking in pregnancy^§^	34 (36.8)	43 (35.0)	0.02 (−0.1; 0.13)
Caucasian^‡^	12 (7.9)	13 (10.2)	−0.02 (−0.09; 0.05)
Education (higher professional)	16 (16.2%)	19 (22.9)	−0.07 (−0.18; 0.05)
Gestational age at birth	266.4 (261.5; 271.2)	277.5 (269.8; 283.6)	−9.91 (−11.85; −7.97)^*^
Male sex	57 (36.5)	53 (39.6)	– 3.1 (−8.0; 1.4)
Birth weight (grams)	2,435 (2,174; 2,660)	2,600 (2,230; 2,850)	−134 (−221; −46)^*^
Birth weight <10th centile	21 (13.3)	37 (27.8)	−14.5 (−24; −5.0)^*^
Birth weight <3rd centile	103 (65.2)	102 (76.7)	−11.5 (−22.0; −1.0)^*^
Composite adverse neonatal outcome^&^	7 (4.9)	7 (6.0)	−1.1 (−7; 4)

* p <0.001.

*Table shows median [(IQR: 25th to 75th percentile) or number (%)]*.

*Data were compared using the Student t-test, chi-square, or Fisher exact test*.

† *n = 141 for induction; n = 122 for expectant management*.

§ *n = 144 for induction; n = 123 for expectant management*.

‡ *n = 151 for induction; n = 127 for expectant management*.

& *n = 143 for induction; n = 116 for expectant management*.

The response rate was 53% (*n* = 526) (Figure 1). Of these patients, 292 were in the “randomized” group. For 487 children, at least 1 measurement of weight was available; for 458 children, 5 or more weight measurements were available; and for 71 children, 10 weight measurements were available. At 2 years of age, height measurement of 98 children and weight measurement of 100 children were available.

**Figure 2:**
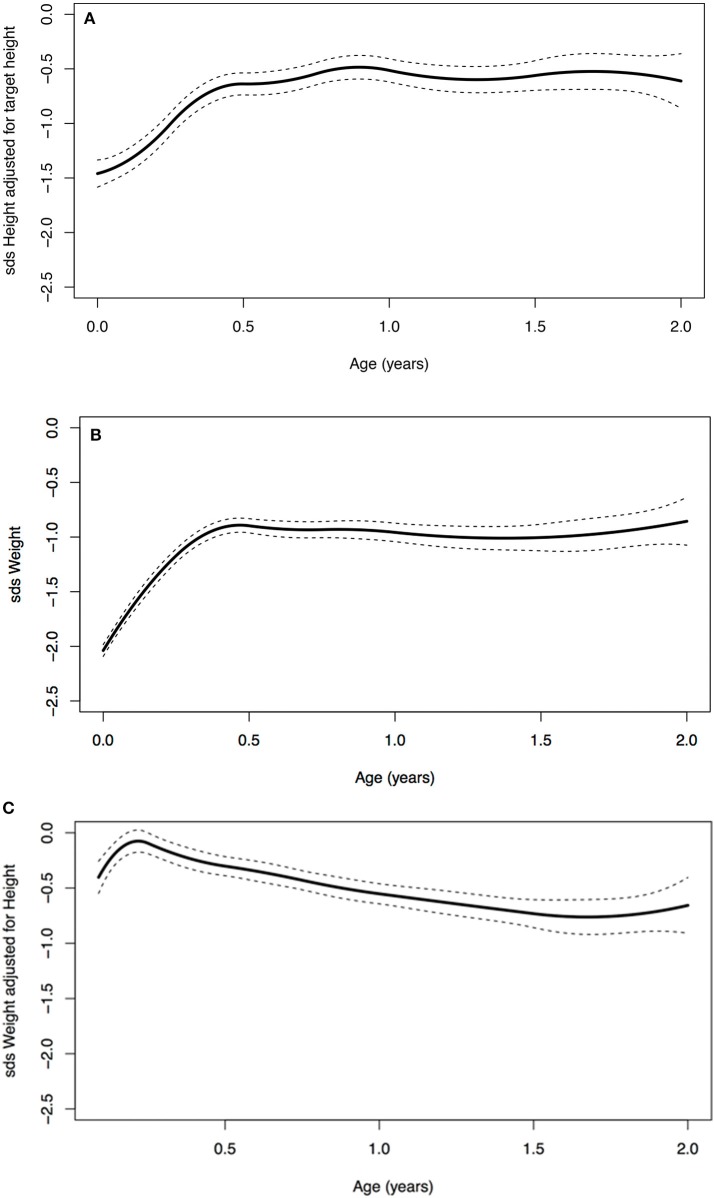
**(A)** Age vs. standard deviation score (SDS) height adjusted for target height, **(B)** SDS weight, and **(C)** SDS weight for height.

A significant increase in SDS in the first 2 years of life was seen (Table 3). At birth, the average SDS was −1.6 for height and −2.0 for weight in the entire group. At 2 years of age, the average SDS was −0.9 [mean increase from birth 0.7 (95% CI: 0.2; 1.0)] for height and −1.0 [mean increase from birth 1.0 (95% CI: 0.7; 1.2)] for weight (Table 3 and Figure 2).

**Table 3:** Age, height, weight, and standard deviation score (SDS) for height, weight, weight for height, height SDS below target height, and difference between SDS in height and weight from birth.

**Age (months)**	**Height (cm) (*n*)**	**Weight(g) (*n*)**	**SDS height**	**SDS weight**	**SDS weight for height (*n*)**	**SDS height-target height (*n*)**	**Difference in SDS height from birth (*n*)**	**95% CI**	**Difference in SDS weight from birth (*n*)**	**95% CI**
Birth	46.4 ± 2.7 (236)	2,493 ± 397 (526)	−1.6 ± 1.3	−2.0 ± 0.86	NA	−1.4 ± 1.4	NA		NA	
1	50.7 ± 2.4 (370)	3,411 ± 476 (392)	−1.7 ± 1.1	−1.8 ± 1.0	−0.5 ± 1.1	−1.5 ± 1.2	0.2 (*n* = 161)	−0.4; 0.03	0.2 (*n* = 388)	0.1; 0.3;
3	57.7 ± 2.7 (419)	5,074 ± 685 (433)	−1.2 ± 1.1	−1.1 ± 1.0	−0.2 ± 1.2	– 0.9 ± 1.1	0.4 (*n* = 188)	0.2; 0.6	0.9 (*n* = 429)	0.8; 1.0
6	64.7 ± 2.8 (439)	6,790 ± 825 (451)	−1.0 ± 1.1	−1.0 ± 1.1	−0.3 ± 1.1	−0.7 ± 1.2	0.6 (*n* = 202)	0.5; 0.8	1.1 (*n* = 446)	1.0; 1.2
9	69.9 ± 2.8 (355)	8,037 ± 937 (360)	−0.9 ± 1.1	– 0.9 ± 1.1	−0.4 ± 1.0	−0.5 ± 1.0	0.8 (*n* = 159)	0.6; 0.9	1.1 (*n =* 356)	1.0; 1.2
12	72.8 ± 3.0 (296)	8,669 ± 1,103(301)	−0.8 ± 1.1	−1.0 ± 1.1	−0.5 ± 1.1	−0.5± 1.1	0.7 (*n* = 148)	0.5; 0.9	1.0 (*n* = 299)	0.9; 1.2
18	80.1 ± 3.3 (190)	10,101 ± 1,103 (192)	−0.9 ± 1.1	−1.1 ±−0.96	−0.8 ± 0.9	−0.5 ± 1.0	0.7 (*n* = 87)	0.4; 1.0	1.0 (*n* = 191)	0.8; 1.1
24	85.3 ± 4.2 (98)	11,362 ± 1,513 (100)	−0.9 ± 1.3	−1.0 ± 1.2	−0.7 ± 1.1	– 0.6 ± 1.2	0.6 (*n* = 43)	0.2; 1.0	0.9 (*n* = 99)	0.7; 1.2

*Table shows mean (n) and standard deviation or 95% CI*.

**Figure 3:**
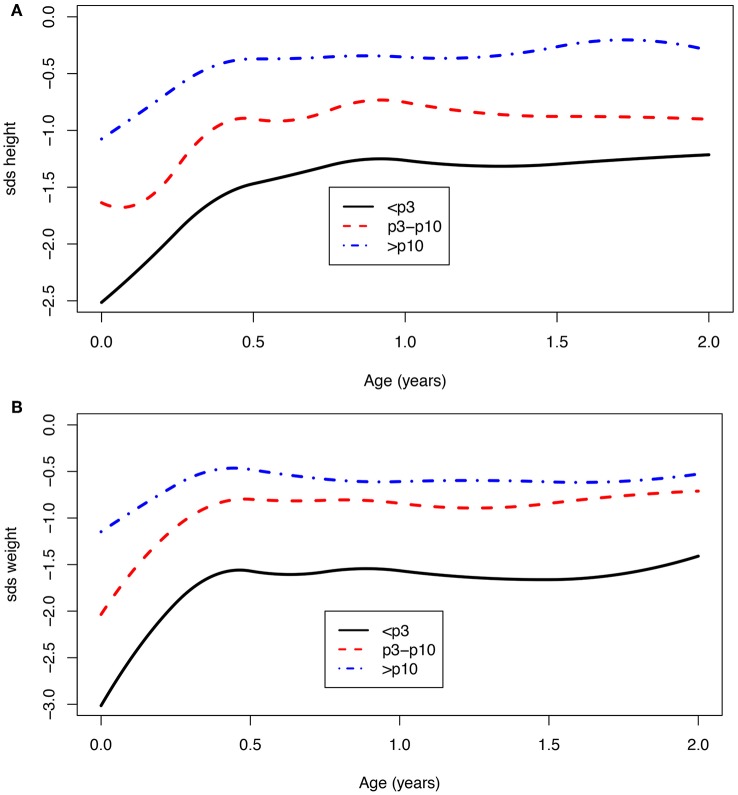
**(A,B)** Age vs. SDS height and SDS weight for a birth weight below the 3rd, between 3rd and 10th, and above the 10th centile.

Children with a birth weight below the 10th centile (<*p*10) were born with a mean SDS of −1.8 for height (*n* = 171) and −2.4 for weight (*n* = 382), and at 2 years of age, the SDS was −1.1 for height (*n* = 67) and −1.2 for weight (*n* = 69). For children who were born with a birth weight that equals or was above the 10th centile (>*p*10), the mean SDS at birth was −1.1 for height (*n* = 63) and −1.2 for weight (*n* = 141), and at 2 years of age, −0.4 for height (*n* = 30) and −0.6 for weight (*n* = 30). Children born with a birth weight <*p*10 showed significantly more catch-up growth in weight at age 1 [mean difference −0.8 SDS (95% CI: −1.1; −0.5)] as well as at age 2 [mean difference −0.7 SDS (95% CI: −1.2; −0.2)] when compared to children born with a birth weight >*p*10 (Figure 3). The same effect was seen for children with a birth weight below the third centile (Figure 3). These children remain lower in SDS height and weight compared to the children born with a birth weight above the 3rd and 10th centiles.

Girls had lower weight SDS at birth [mean difference 0.3 SDS (95% CI: 0.1; 0.4)] and show more catch-up growth in weight particularly in the first month compared to boys (results in Supplementary Material).

**Figure 4:**
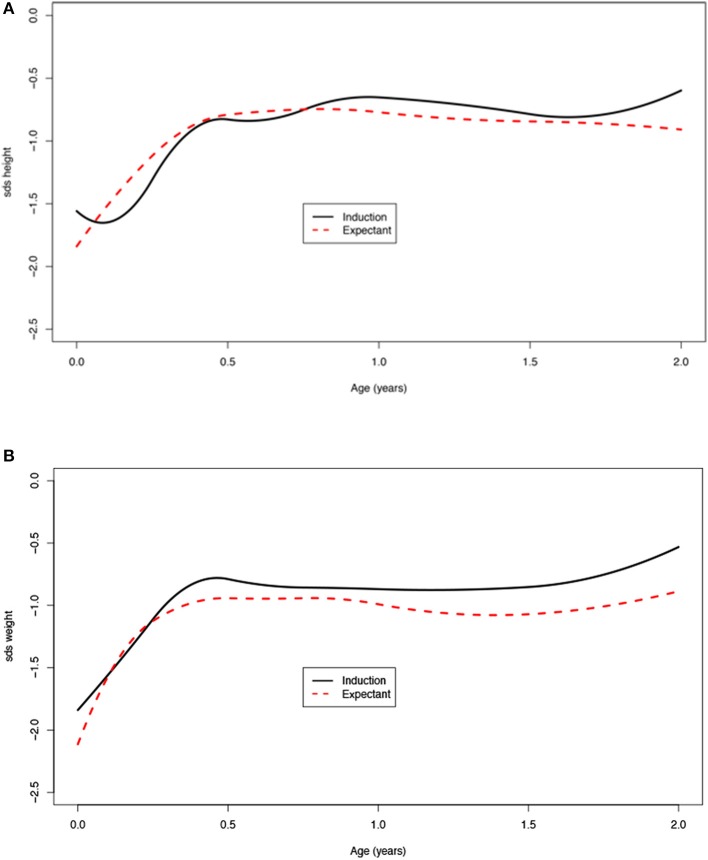
**(A,B)** Age vs. SDS height and weight for induction of labor vs. expectant management.

Children born after obstetric policy of EM compared to children born after IoL showed significantly more catch-up growth in height [mean difference 0.7 (95% CI: 0.2; 1.3)] and weight [mean difference 0.5 (95% CI: 0.3; 0.7)] during the first month after birth. Thereafter, the growth patterns of the two groups were similar (Figure 4, Table 4).

**Table 4:** Mean SDS height corrected for target height (TH), SDS weight, and catch-up growth (SDS weight–SDS birth) (± standard deviation) at different ages compared between children randomized for induction or expectant monitoring.

		**Induction**			**Expectant management**			**Mean difference (95% CI)**	***p*-value**
		***N***	**Mean**	**SD**	***N***	**Mean**	**SD**		
SDS height-TH (SD)	1 month	97	−1.6	1.2	83	−1.2	1.1	−0.4 (−0.8;−0.1)	0.014
	6 months	112	−0.7	1.1	103	−0.5	1.2	−0.2 (−0.5; 0.1)	0.251
	9 months	92	−0.4	1.0	84	−0.3	0.9	−0.1 (−0.4; 0.2)	0.418
	12 months	78	−0.4	0.8	62	−0.4	1.3	0.1 (−0.3; 0.4)	0.759
	18 months	48	−0.5	0.8	51	−0.5	1.1	0.0 (−0.4; 0.4)	0.917
SDS birth weight	Birth	148	−1.8	0.7	121	−2.1	0.9	0.3 (0.1;0.5)	0.005
	1 month	115	−1.9	1.0	97	−1.7	1.0	−0.2 (−0.4; 0.1)	0.238
	3 months	125	−1.0	0.9	104	−1.1	0.9	0.1 (−0.2; 0.3)	0.638
	6 months	129	−0.9	1.0	112	−1.0	1.1	0.1 (−0.2; 0.4)	0.424
	9 months	105	−0.8	1.0	92	−0.9	1.0	0.1 (−0.2; 0.4)	0.600
	12 months	92	−0.9	0.9	66	−1.1	1.3	0.2 (−0.2; 0.5)	0.338
	18 months	57	−1.0	0.8	53	−1.0	0.9	0.0 (−0.3; 0.4)	0.879
Catch-up growth	1 month	115	0.1	0.9	97	−0.4	0.8	0.5 (0.3; 0.7)	0.000
SDS weight–SDS birth	3 months	125	−0.8	1.0	104	−1.0	0.9	0.2 (0.0; 0.5)	0.069
	6 months	129	−1.0	1.1	112	−1.1	1.1	0.2 (−0.1; 0.5)	0.205
	9 months	105	−1.0	1.2	92	−1.2	1.1	0.2 (−0.2; 0.5)	0.322
	12 months	92	−0.9	1.2	66	−1.0	1.1	0.1 (−0.3; 0.4)	0.667
	18 months	57	−0.9	0.8	53	−1.1	1.1	0.3 (−0.1; 0.6)	0.168

Children with adverse neonatal outcome had lower SDS birth weight. We did not observe significantly more catch-up growth in this group, but numbers were low (Supplementary Material). Smoking during pregnancy resulted in 0.3 SDS (95% CI: −0.5; −0.1) lower birth weight in comparison to nonsmoking; a significant difference was seen in catch-up growth in the first 6 months after birth between children from smoking vs. nonsmoking mothers: after 6 months, the SDS scores were very similar in both groups (Supplementary Material). We did not see any effect of breastfeeding (Supplementary Material).

Complete catch-up growth in height was seen in 86% (*n* = 311) of children, and 84% (*n* = 335) complete catch-up in weight after 1 year. The mean target height SDS for the entire group was −0.3 SDS; the mean observed SD height score at 2 years of age was −0.9 SD, which is still significantly lower than the expected target height SD (*p* = 0.000).

When looking at neurodevelopmental and behavioral outcome problems at 2 years of age found by the ASQ and CBCL, we found that children with an abnormal score on the ASQ and were 6 and 12 months of age showed more catch-up in weight and were more below their target height when compared to the children with a normal ASQ (Table 5). We found no difference in catch-up height or weight in children with an adverse outcome for the CBCL (Supplementary Material). In a multivariate logistic model, birth weight SDS had a strong association with an abnormal outcome of ASQ [a decrease of 1 birth weight SDS yielded a 2.6 (95% CI: 1.8; 3.5) times higher odds on an abnormal ASQ score]. Also, a moderate effect of gestational age [a 1-week longer gestational age increased the odds on an abnormal ASQ score by 1.21 (95% CI: 0.98; 1.50)] was found. Adding growth variables during the first month did not significantly improve the model and did not remarkably change these odds ratios.

**Table 5:** Mean SDS height corrected for target height (TH), SDS birth weight and catch up growth (SDS weight-SDS birth) (± standard deviation) at different ages compared between neurodevelopmental outcomes at age 2.

	**Abnormal ASQ**			
	**Yes**	**No**	**Difference in mean (95% CI)**	***p*-value**
**SDS height-TH (SD)**				
6 months (*n* = 376)	−1.1 (1.1)	−0.6 (1.1)	−0.5 (−0.8; 0.2)	0.002
12 months (*n* = 268)	−1.1 (1.1)	−0.4 (1.0)	−0.7 (−1.0; −0.3)	0.002
18 months (*n* = 355)	−0.9 (1.2)	−0.5 (0.9)	−0.4 (−0.9; 0.2)	0.1
SDS Birth weight	−2.7 (0.8)	−1.9 (0.8)	−0.8 (−1.0; −0.6)	<0.001
**Catch-up growth SDS weight-SDS birth**				
6 months (*n* = 443)	1.3 (0.9)	1.0 (1.0)	0.3 (0.04; 0.6)	0.03
12 months (*n* = 355)	1.3 (0.9)	1.0 (1.1)	0.3 (0.005; 0.7)	0.047
18 months (*n* = 188)	1.2 (1.3)	0.9 (1.0)	0.3 (−0.2; 0.7)	0.3

## Discussion

Children born after EM of labor in FGR were more severely growth restricted at birth than children born after IoL (16), and these children exhibited more catch-up growth during the first month after birth. Also, growth-restricted children with a birth weight below the 10th centile showed more catch-up growth than those born with birth weights above the 10th centile. Catch-up growth occurs fastest in the first months after birth.

The fact that children born with a birth weight below the 10th centile showed significantly more catch-up growth than children born with a birth weight above the 10th centile indicates that more of these children were truly growth restricted and therefore might be at greater risk of developing metabolic syndrome in later life (7–9, 24). Children born after an EM also showed more catch-up growth during the first month after birth than children born after IoL. The difference in gestational age at birth between these children is on average 10 days. One could argue that keeping a fetus in an undernourished intrauterine environment for 10 days longer leads to more serious growth restriction and, in turn, also to more catch-up growth. It is therefore likely that the antenatal decision either to actively end the undernourished status by inducing labor or to “wait and see”—an EM—influences long-term outcome and puts these children at a greater risk for metabolic and cardiovascular problems in later life. The long-term effects in relation to catch-up growth (i.e., during the first month, first years, etc.). remain unclear, and therefore, the long-term effects of catch-up growth during the first month after birth are undefined.

In children with neurodevelopmental problems at 2 years of age, more catch-up was seen in weight and height when compared to children with a normal neurodevelopmental outcome. This effect, however, disappears when correcting for weight SDS and gestational age at birth. We previously found that birth weight centile is the most important factor of influence on neurodevelopmental outcome at age 2, reflecting the dose effect of the severity of FGR (placental insufficiency) (17).

At 2 years of age, the majority of children of the DIGITAT cohort have height higher than 1.6 SD below their target height range and weight above −2 SDS, indicating that catch-up growth is, for the greatest part, complete (12). However, height and height SDS corrected for mid-parental height (target height SDS) and weight SDS are still significantly lower than expected in these children based on the norm population.

Our study is the first study to compare the effects of IoL with EM on postnatal catch-up growth in FGR. Previous studies have shown that children born after FGR exhibit catch-up growth in height and weight (1–3, 5, 6, 9, 12), but none of these studies studied catch-up growth in relation to obstetric policies to growth at 2 years of age. Another unique aspect of our study is that we included children based on suspected FGR and not based on actual birth weight, making comparisons with children with a birth weight above the 10th possible.

A weakness of our study is a possible response bias as data were obtained through postal inquiries and data were completed by the parents. We do, however, believe our measurements are reliable, as the children were measured by child health care professionals during routine scheduled child health care visits and parents were asked to provide us with those measurements. Respondents (mothers of the children) were slightly older, smoked less, higher educated, and more frequently Caucasian. Whereas, the percentage of growth-restricted children was comparable between non-respondents and respondents, there might have been more healthy life and feeding styles in respondent families; we do not know, however, to which direction our results would shift if the total sample would have been available. Unfortunately, our response rate was only around 50%, and the number of measurements at the age of 2 years is much lower. Within the group of respondents, we do not expect the group of children of whom we have measurements at age 2 to differ from the group of children without measurements at age 2. We suspect that missing values at 2 years are due to logistical reasons: the questionnaires were sent out around the age of 2 years (22–26 months of age) when a considerable group of parents had not yet been to the child health clinic for the scheduled 2-year visit and therefore did not have the measurements. We, therefore, do not expect a bias in our results, but it does influence our sample sizes.

The timing of IoL remains difficult however, as IoL before 38 weeks' gestation increases risks of complications due to late prematurity (25, 26). There are no known applicable biophysical markers that reflect maturation of fetuses; the best timing of induction depends, for the greatest part, on clinical assessment. We hypothesize that IoL in suspected FGR is optimal around 38 weeks (25), minimizing negative effects of being born late premature and hopefully preventing the risks from FGR and catch-up growth.

## Conclusion

The majority of children born after FGR at term exhibit catch-up growth after birth and show complete catch-up growth after 2 years. After an EM policy, more children are severely growth restricted and show more catch-up growth during the first month after birth. A lower birth weight percentile was found as the most important factor influencing adverse neurodevelopmental outcome at age 2.

## Ethics Statement

This study has been approved by the ethics committee of the Leiden University Medical Center (Ref. No. P04.210).

## Author Contributions

LW, SC, and KBo performed the analyses, drafted the initial manuscript, and approved the final manuscript as submitted. SS conceptualized and designed the study, interpreted the data, and critically reviewed the manuscript. JP, AW-L, HB, SG, FD, MP, KBl, FR, JL, and BM critically reviewed the manuscript. All authors have seen and approved the final manuscript.

### Conflict of Interest Statement

The authors declare that the research was conducted in the absence of any commercial or financial relationships that could be construed as a potential conflict of interest. The handling Editor declared a past co-authorship with one of the authors SS.

## Abbreviations

ASQ: Ages and Stages Questionairre
CBCL: Child Behavior Checklist
CI: confidence interval
DIGITAT: Disproportionate Intrauterine Growth Restriction Trial at Term
IQR: interquartile range
FGR: fetal growth restriction
SD: standard deviation
SDS: standard deviation score.
