# Supplementary material for: Postnatal Catch-Up Growth After Suspected Fetal Growth Restriction at Term
Source: Front Endocrinol (Lausanne). 2019 Jun 20;10:274. doi: 10.3389/fendo.2019.00274 (PMC6598620; doi:10.3389/fendo.2019.00274)
Supplement: Supplementary file 1 [file Table_1.DOCX]

**Table A1: Mean SDS height corrected for target height (TH), SDS weight and catch up growth ( SDS weight-SDS birth) (± standard deviation) at different ages compared between children randomized for induction or expectant monitoring**

|  |  | Induction | | | expectant management | | |  | Mean Difference | 95% Confidence Interval of the Difference | | p-value |
| --- | --- | --- | --- | --- | --- | --- | --- | --- | --- | --- | --- | --- |
|  |  | N | Mean | Std. Deviation | N | Mean | Std. Deviation |  |  | Lower | Upper |  |
| **SDS height-TH (SD)** | 1 month | 97 | -1.6 | 1.2 | 83 | -1.2 | 1.1 |  | -0.4 | -0.8 | -0.1 | .014 |
|  | 6 months | 112 | -0.7 | 1.1 | 103 | -0.5 | 1.2 |  | -0.2 | -0.5 | 0.1 | .251 |
|  | 9 months | 92 | -0.4 | 1.0 | 84 | -0.3 | 0.9 |  | -0.1 | -0.4 | 0.2 | .418 |
|  | 12 months | 78 | -0.4 | 0.8 | 62 | -0.4 | 1.3 |  | 0.1 | -0.3 | 0.4 | .759 |
|  | 18 months | 48 | -0.5 | 0.8 | 51 | -0.5 | 1.1 |  | 0.0 | -0.4 | 0.4 | .917 |
| **SDS Birth weight** | Birth | 148 | -1.8 | 0.7 | 121 | -2.1 | 0.9 |  | 0.3 | 0.1 | 0.5 | .005 |
|  | 1 month | 115 | -1.9 | 1.0 | 97 | -1.7 | 1.0 |  | -0.2 | -0.4 | 0.1 | .238 |
|  | 3 months | 125 | -1.0 | 0.9 | 104 | -1.1 | 0.9 |  | 0.1 | -0.2 | 0.3 | .638 |
|  | 6 months | 129 | -0.9 | 1.0 | 112 | -1.0 | 1.1 |  | 0.1 | -0.2 | 0.4 | .424 |
|  | 9 months | 105 | -0.8 | 1.0 | 92 | -0.9 | 1.0 |  | 0.1 | -0.2 | 0.4 | .600 |
|  | 12 months | 92 | -0.9 | 0.9 | 66 | -1.1 | 1.3 |  | 0.2 | -0.2 | 0.5 | .338 |
|  | 18 months | 57 | -1.0 | 0.8 | 53 | -1.0 | 0.9 |  | 0.0 | -0.3 | 0.4 | .879 |
| **Catch-up growth** | 1 month | 115 | 0.1 | 0.9 | 97 | -0.4 | 0.8 |  | 0.5 | 0.3 | 0.7 | .000 |
| **SDS weight-SDS birth** | 3 months | 125 | -0.8 | 1.0 | 104 | -1.0 | 0.9 |  | 0.2 | 0.0 | 0.5 | .069 |
|  | 6 months | 129 | -1.0 | 1.1 | 112 | -1.1 | 1.1 |  | 0.2 | -0.1 | 0.5 | .205 |
|  | 9 months | 105 | -1.0 | 1.2 | 92 | -1.2 | 1.1 |  | 0.2 | -0.2 | 0.5 | .322 |
|  | 12 months | 92 | -0.9 | 1.2 | 66 | -1.0 | 1.1 |  | 0.1 | -0.3 | 0.4 | .667 |
|  | 18 months | 57 | -0.9 | 0.8 | 53 | -1.1 | 1.1 |  | 0.3 | -0.1 | 0.6 | .168 |

**Table A2: Mean SDS height corrected for target height (TH), SDS weight and catch up growth ( SDS weight-SDS birth) (± standard deviation) at different ages compared between children below and above the 10^th^ birthweight percentile**

|  |  | < p10 | | | > p10 | | |  | Mean Difference | 95% Confidence Interval of the Difference | | p-value |
| --- | --- | --- | --- | --- | --- | --- | --- | --- | --- | --- | --- | --- |
|  |  | N | Mean | Std. Deviation | N | Mean | Std. Deviation |  |  | Lower | Upper |  |
| **SDS height-TH (SD)** | 1 month | 224 | -1.7 | 1.1 | 84 | -0.9 | 1.2 |  | -0.7 | -1.0 | -0.4 | .000 |
|  | 6 months | 278 | -0.8 | 1.2 | 99 | -0.4 | 1.0 |  | -0.4 | -0.7 | -0.2 | .002 |
|  | 9 months | 222 | -0.6 | 1.1 | 78 | -0.2 | 0.7 |  | -0.4 | -0.7 | -0.1 | .002 |
|  | 12 months | 182 | -0.6 | 1.2 | 70 | -0.3 | 0.8 |  | -0.3 | -0.6 | 0.0 | .039 |
|  | 18 months | 122 | -0.6 | 0.9 | 46 | -0.3 | 1.1 |  | -0.3 | -0.7 | 0.0 | .047 |
| **SDS Birth weight** | Birth | 357 | -2.4 | 0.7 | 129 | -1.2 | 0.5 |  | -1.2 | -1.4 | -1.1 | .000 |
|  | 1 month | 281 | -2.1 | 1.0 | 105 | -1.1 | 0.9 |  | -1.0 | -1.2 | -0.8 | .000 |
|  | 3 months | 307 | -1.3 | 1.0 | 120 | -0.7 | 0.8 |  | -0.7 | -0.9 | -0.5 | .000 |
|  | 6 months | 329 | -1.1 | 1.1 | 116 | -0.5 | 0.8 |  | -0.6 | -0.8 | -0.4 | .000 |
|  | 9 months | 260 | -1.1 | 1.1 | 94 | -0.5 | 0.8 |  | -0.5 | -0.7 | -0.3 | .000 |
|  | 12 months | 214 | -1.1 | 1.1 | 84 | -0.7 | 0.9 |  | -0.4 | -0.7 | -0.1 | .003 |
|  | 18 months | 140 | -1.2 | 0.9 | 51 | -0.8 | 1.0 |  | -0.4 | -0.7 | -0.1 | .011 |
| **Catch-up growth** | 1 month | 281 | -0.2 | 0.9 | 105 | -0.1 | 0.8 |  | -0.1 | -0.3 | 0.1 | .163 |
| **SDS weight-SDS birth** | 3 months | 307 | -1.0 | 0.9 | 120 | -0.5 | 0.9 |  | -0.5 | -0.7 | -0.3 | .000 |
|  | 6 months | 329 | -1.2 | 1.0 | 116 | -0.6 | 0.9 |  | -0.6 | -0.8 | -0.4 | .000 |
|  | 9 months | 260 | -1.3 | 1.0 | 94 | -0.6 | 0.9 |  | -0.6 | -0.9 | -0.4 | .000 |
|  | 12 months | 214 | -1.3 | 1.1 | 84 | -0.4 | 1.0 |  | -0.8 | -1.1 | -0.5 | .000 |
|  | 18 months | 140 | -1.2 | 1.0 | 51 | -0.4 | 0.9 |  | -0.8 | -1.1 | -0.5 | .000 |

**Table A3: Mean SDS height corrected for target height (TH), SDS weight and catch up growth ( SDS weight-SDS birth) (± standard deviation) at different ages compared between boys and girls.**

|  |  | male | | | female | | |  | Mean Difference | 95% Confidence Interval of the Difference | | p-value |
| --- | --- | --- | --- | --- | --- | --- | --- | --- | --- | --- | --- | --- |
|  |  | N | Mean | Std. Deviation | N | Mean | Std. Deviation |  |  | Lower | Upper |  |
| **SDS height-TH (SD)** | 1 month | 114 | -1.5 | 1.3 | 198 | -1.5 | 1.1 |  | -0.1 | -0.3 | 0.2 | .624 |
|  | 6 months | 144 | -0.8 | 1.4 | 235 | -0.7 | 1.0 |  | -0.1 | -0.4 | 0.1 | .291 |
|  | 9 months | 116 | -0.5 | 1.2 | 188 | -0.5 | 0.9 |  | 0.0 | -0.3 | 0.2 | .889 |
|  | 12 months | 94 | -0.7 | 1.2 | 160 | -0.5 | 1.0 |  | -0.2 | -0.5 | 0.1 | .119 |
|  | 18 months | 66 | -0.6 | 1.2 | 102 | -0.5 | 0.9 |  | -0.1 | -0.4 | 0.2 | .602 |
| **SDS Birth weight** | Birth | 186 | -1.9 | 0.9 | 299 | -2.2 | 0.8 |  | 0.3 | 0.1 | 0.4 | .001 |
|  | 1 month | 139 | -2.0 | 1.1 | 251 | -1.8 | 1.0 |  | -0.2 | -0.4 | 0.0 | .065 |
|  | 3 months | 163 | -1.2 | 1.1 | 268 | -1.1 | 1.0 |  | -0.2 | -0.4 | 0.0 | .140 |
|  | 6 months | 170 | -1.1 | 1.1 | 278 | -0.9 | 1.0 |  | -0.1 | -0.4 | 0.1 | .153 |
|  | 9 months | 136 | -1.0 | 1.1 | 222 | -0.9 | 1.0 |  | -0.1 | -0.3 | 0.1 | .479 |
|  | 12 months | 108 | -1.1 | 1.1 | 192 | -1.0 | 1.1 |  | -0.1 | -0.4 | 0.1 | .392 |
|  | 18 months | 76 | -1.1 | 1.0 | 115 | -1.1 | 0.9 |  | 0.0 | -0.3 | 0.2 | .742 |
| **Catch-up growth** | 1 month | 139 | 0.2 | 0.8 | 249 | -0.4 | 0.8 |  | 0.5 | 0.4 | 0.7 | .000 |
| **SDS weight-SDS birth** | 3 months | 163 | -0.7 | 0.9 | 266 | -1.0 | 0.9 |  | 0.4 | 0.2 | 0.6 | .000 |
|  | 6 months | 170 | -0.8 | 1.0 | 276 | -1.2 | 1.0 |  | 0.4 | 0.2 | 0.6 | .000 |
|  | 9 months | 136 | -0.9 | 1.1 | 220 | -1.3 | 1.0 |  | 0.4 | 0.1 | 0.6 | .002 |
|  | 12 months | 108 | -0.7 | 1.2 | 191 | -1.2 | 1.1 |  | 0.4 | 0.2 | 0.7 | .001 |
|  | 18 months | 76 | -0.8 | 1.1 | 115 | -1.1 | 1.0 |  | 0.3 | 0.0 | 0.6 | .065 |

**Table A4: Mean SDS height corrected for target height (TH), SDS weight and catch up growth ( SDS weight-SDS birth) (± standard deviation) at different ages compared between children with and without adverse neonatal outcomes**

|  |  | No adverse outcomes | | | adverse outcomes | | |  | Mean Difference | 95% Confidence Interval of the Difference | | p-value |
| --- | --- | --- | --- | --- | --- | --- | --- | --- | --- | --- | --- | --- |
|  |  | N | Mean | Std. Deviation | N | Mean | Std. Deviation |  |  | Lower | Upper |  |
| **SDS height-TH (SD)** | 1 month | 264 | -1.5 | 1.2 | 13 | -1.9 | 1.5 |  | 0.5 | -0.2 | 1.1 | .165 |
|  | 6 months | 319 | -0.7 | 1.2 | 18 | -1.1 | 1.2 |  | 0.4 | -0.1 | 1.0 | .117 |
|  | 9 months | 258 | -0.4 | 1.0 | 11 | -0.4 | 1.1 |  | 0.0 | -0.7 | 0.6 | .932 |
|  | 12 months | 217 | -0.5 | 1.1 | 9 | -0.6 | 1.5 |  | 0.0 | -0.7 | 0.8 | .925 |
|  | 18 months | 139 | -0.5 | 1.0 | 11 | -1.0 | 0.9 |  | 0.5 | -0.1 | 1.1 | .113 |
| **SDS Birth weight** | Birth | 408 | -2.0 | 0.8 | 26 | -2.7 | 1.1 |  | 0.7 | 0.3 | 1.0 | .000 |
|  | 1 month | 332 | -1.8 | 1.0 | 16 | -2.4 | 1.1 |  | 0.6 | 0.1 | 1.1 | .024 |
|  | 3 months | 363 | -1.1 | 1.0 | 23 | -1.7 | 0.9 |  | 0.6 | 0.2 | 1.0 | .007 |
|  | 6 months | 380 | -0.9 | 1.1 | 22 | -1.3 | 1.0 |  | 0.4 | -0.1 | 0.9 | .096 |
|  | 9 months | 305 | -0.9 | 1.0 | 14 | -1.2 | 0.8 |  | 0.3 | -0.2 | 0.9 | .252 |
|  | 12 months | 257 | -1.0 | 1.1 | 12 | -1.4 | 1.0 |  | 0.4 | -0.3 | 1.0 | .247 |
|  | 18 months | 157 | -1.1 | 1.0 | 14 | -1.3 | 1.0 |  | 0.2 | -0.3 | 0.7 | .464 |
| **Catch-up growth** | 1 month | 330 | -0.2 | 0.8 | 16 | -0.2 | 0.8 |  | 0.1 | -0.4 | 0.5 | .791 |
| **SDS weight-SDS birth** | 3 months | 361 | -0.9 | 0.9 | 23 | -1.0 | 1.0 |  | 0.1 | -0.3 | 0.4 | .791 |
|  | 6 months | 378 | -1.1 | 1.0 | 22 | -1.4 | 1.0 |  | 0.3 | -0.2 | 0.7 | .206 |
|  | 9 months | 303 | -1.2 | 1.0 | 14 | -1.2 | 1.1 |  | 0.1 | -0.5 | 0.7 | .734 |
|  | 12 months | 256 | -1.0 | 1.1 | 12 | -1.3 | 1.3 |  | 0.2 | -0.5 | 0.9 | .538 |
|  | 18 months | 157 | -0.9 | 1.0 | 14 | -1.6 | 1.2 |  | 0.7 | 0.1 | 1.3 | .023 |

**Table A5: Mean SDS height corrected for target height (TH), SDS weight and catch up growth ( SDS weight-SDS birth) (± standard deviation) at different ages compared between children who received and who did not receive breast feeding**

|  |  | Started breastfeeding | | | No breastfeeding | | |  | Mean Difference | 95% Confidence Interval of the Difference | | p-value |
| --- | --- | --- | --- | --- | --- | --- | --- | --- | --- | --- | --- | --- |
|  |  | N | Mean | Std. Deviation | N | Mean | Std. Deviation |  |  | Lower | Upper |  |
| **SDS height-TH (SD)** | 1 month | 191 | -1.5 | 1.2 | 119 | -1.5 | 1.3 |  | 0.0 | -0.3 | 0.3 | .948 |
|  | 6 months | 226 | -0.8 | 1.2 | 149 | -0.6 | 1.1 |  | -0.1 | -0.4 | 0.1 | .319 |
|  | 9 months | 184 | -0.5 | 1.1 | 118 | -0.3 | 0.9 |  | -0.2 | -0.4 | 0.0 | .099 |
|  | 12 months | 157 | -0.5 | 1.0 | 95 | -0.6 | 1.2 |  | 0.0 | -0.2 | 0.3 | .728 |
|  | 18 months | 96 | -0.5 | 0.9 | 68 | -0.5 | 1.0 |  | 0.0 | -0.3 | 0.3 | .966 |
| **SDS Birth weight** | Birth | 294 | -2.1 | 0.9 | 190 | -2.1 | 0.8 |  | 0.0 | -0.2 | 0.2 | .978 |
|  | 1 month | 238 | -1.8 | 1.1 | 150 | -1.9 | 1.0 |  | 0.1 | -0.2 | 0.3 | .636 |
|  | 3 months | 257 | -1.2 | 1.1 | 171 | -1.1 | 0.9 |  | -0.1 | -0.3 | 0.1 | .591 |
|  | 6 months | 263 | -1.0 | 1.1 | 181 | -0.9 | 1.1 |  | -0.1 | -0.3 | 0.1 | .240 |
|  | 9 months | 213 | -1.0 | 1.1 | 143 | -0.8 | 0.9 |  | -0.2 | -0.4 | 0.0 | .115 |
|  | 12 months | 181 | -1.0 | 1.1 | 117 | -1.1 | 1.1 |  | 0.1 | -0.2 | 0.3 | .609 |
|  | 18 months | 109 | -1.2 | 0.9 | 78 | -1.0 | 1.0 |  | -0.1 | -0.4 | 0.2 | .372 |
| **Catch-up growth** | 1 month | 238 | -0.2 | 0.8 | 148 | -0.2 | 0.9 |  | -0.1 | -0.2 | 0.1 | .530 |
| **SDS weight-SDS birth** | 3 months | 257 | -0.9 | 0.9 | 169 | -0.9 | 0.9 |  | 0.0 | -0.2 | 0.2 | .745 |
|  | 6 months | 263 | -1.0 | 1.0 | 179 | -1.2 | 1.0 |  | 0.1 | -0.1 | 0.3 | .168 |
|  | 9 months | 213 | -1.0 | 1.1 | 141 | -1.3 | 1.0 |  | 0.2 | 0.0 | 0.4 | .050 |
|  | 12 months | 181 | -1.0 | 1.1 | 116 | -1.0 | 1.1 |  | 0.0 | -0.3 | 0.3 | .939 |
|  | 18 months | 109 | -0.9 | 1.0 | 78 | -1.0 | 1.2 |  | 0.1 | -0.2 | 0.4 | .560 |

**Table A6: Mean SDS height corrected for target height (TH), SDS weight and catch up growth ( SDS weight-SDS birth) (± standard deviation) at different ages compared between smoking during pregnagcy and not smoking during pregnancy.**

|  |  | Smoking | | | Non smoking | | |  | Mean Difference | 95% Confidence Interval of the Difference | | p-value |
| --- | --- | --- | --- | --- | --- | --- | --- | --- | --- | --- | --- | --- |
|  |  | N | Mean | Std. Deviation | N | Mean | Std. Deviation |  |  | Lower | Upper |  |
| **SDS height-TH (SD)** | 1 month | 74 | -1.7 | 1.2 | 222 | -1.4 | 1.2 |  | -0.2 | -0.5 | 0.1 | .156 |
|  | 6 months | 99 | -1.0 | 1.1 | 258 | -0.6 | 1.2 |  | -0.4 | -0.6 | -0.1 | .011 |
|  | 9 months | 80 | -0.5 | 0.9 | 209 | -0.4 | 1.1 |  | -0.1 | -0.4 | 0.2 | .442 |
|  | 12 months | 69 | -0.7 | 1.1 | 173 | -0.5 | 1.1 |  | -0.3 | -0.6 | 0.0 | .067 |
|  | 18 months | 47 | -0.7 | 0.9 | 112 | -0.4 | 1.0 |  | -0.2 | -0.6 | 0.1 | .174 |
| **SDS Birth weight** | Birth | 125 | -2.3 | 0.9 | 323 | -2.0 | 0.8 |  | -0.3 | -0.5 | -0.1 | .001 |
|  | 1 month | 88 | -2.0 | 1.0 | 269 | -1.8 | 1.1 |  | -0.2 | -0.5 | 0.1 | .132 |
|  | 3 months | 101 | -1.2 | 1.0 | 292 | -1.1 | 1.1 |  | -0.1 | -0.3 | 0.2 | .485 |
|  | 6 months | 112 | -1.0 | 1.2 | 299 | -1.0 | 1.1 |  | 0.0 | -0.3 | 0.2 | .834 |
|  | 9 months | 88 | -0.8 | 1.0 | 242 | -0.9 | 1.1 |  | 0.1 | -0.2 | 0.4 | .441 |
|  | 12 months | 76 | -1.1 | 1.3 | 201 | -1.0 | 1.1 |  | -0.1 | -0.4 | 0.2 | .626 |
|  | 18 months | 51 | -1.1 | 0.9 | 127 | -1.1 | 1.0 |  | 0.1 | -0.3 | 0.4 | .736 |
| **Catch-up growth** | 1 month | 88 | -0.2 | 0.8 | 267 | -0.2 | 0.9 |  | -0.1 | -0.3 | 0.1 | .459 |
| **SDS weight-SDS birth** | 3 months | 101 | -1.0 | 0.9 | 290 | -0.8 | 0.9 |  | -0.2 | -0.4 | 0.0 | .080 |
|  | 6 months | 112 | -1.3 | 1.1 | 297 | -1.0 | 1.0 |  | -0.3 | -0.6 | -0.1 | .004 |
|  | 9 months | 88 | -1.5 | 1.0 | 240 | -1.0 | 1.0 |  | -0.5 | -0.8 | -0.3 | .000 |
|  | 12 months | 76 | -1.2 | 1.3 | 200 | -1.0 | 1.0 |  | -0.2 | -0.5 | 0.1 | .132 |
|  | 18 months | 51 | -1.2 | 0.9 | 127 | -0.8 | 1.0 |  | -0.4 | -0.7 | 0.0 | .025 |

**Table A7: Mean SDS height corrected for target height (TH), SDS birth weight and catch up growth ( SDS weight-SDS birth) (± standard deviation) at different ages compared between behavioral outcomes at age 2.**

|  |  | CBCL abnormal | | | CBCL Normal | | |  | Mean Difference | 95% Confidence Interval of the Difference | | Sig. (2-tailed) |
| --- | --- | --- | --- | --- | --- | --- | --- | --- | --- | --- | --- | --- |
|  |  | N | Mean | Std. Deviation | N | Mean | Std. Deviation |  |  | Lower | Upper |  |
| **SDS height-TH (SD)** | 1 month | 13 | -2.3 | 1.3 | 297 | -1.4 | 1.2 |  | -0.9 | -1.5 | -0.2 | 0.011 |
|  | 6 months | 23 | -0.9 | 1.2 | 353 | -0.7 | 1.2 |  | -0.2 | -0.7 | 0.2 | 0.336 |
|  | 9 months | 16 | -0.5 | 0.9 | 287 | -0.5 | 1.0 |  | -0.1 | -0.6 | 0.5 | 0.817 |
|  | 12 months | 15 | -0.6 | 1.1 | 237 | -0.5 | 1.1 |  | -0.1 | -0.6 | 0.5 | 0.815 |
|  | 18 months | 12 | -0.5 | 1.0 | 153 | -0.5 | 1.0 |  | 0.0 | -0.5 | 0.6 | 0.872 |
| **SDS Birth weight** | Birth | 30 | -2.2 | 0.9 | 487 | -2.1 | 0.9 |  | -0.2 | -0.5 | 0.1 | 0.237 |
|  | 1 month | 19 | -2.2 | 1.1 | 369 | -1.8 | 1.0 |  | -0.4 | -0.9 | 0.1 | 0.092 |
|  | 3 months | 24 | -1.5 | 1.0 | 405 | -1.1 | 1.0 |  | -0.4 | -0.8 | 0.0 | 0.078 |
|  | 6 months | 28 | -1.2 | 1.1 | 417 | -1.0 | 1.1 |  | -0.2 | -0.6 | 0.2 | 0.315 |
|  | 9 months | 19 | -0.9 | 0.9 | 338 | -0.9 | 1.0 |  | 0.0 | -0.4 | 0.5 | 0.867 |
|  | 12 months | 19 | -0.9 | 0.9 | 279 | -1.0 | 1.1 |  | 0.2 | -0.3 | 0.7 | 0.516 |
|  | 18 months | 14 | -1.2 | 0.9 | 174 | -1.1 | 1.0 |  | -0.1 | -0.6 | 0.5 | 0.790 |
| **Catch-up growth** | 1 month | 19 | 0.2 | 0.7 | 367 | -0.2 | 0.9 |  | 0.4 | 0.0 | 0.8 | 0.070 |
| **SDS weight-SDS birth** | 3 months | 24 | -0.8 | 0.8 | 403 | -0.9 | 0.9 |  | 0.1 | -0.3 | 0.5 | 0.586 |
|  | 6 months | 28 | -1.0 | 0.9 | 415 | -1.1 | 1.0 |  | 0.1 | -0.3 | 0.5 | 0.735 |
|  | 9 months | 19 | -1.4 | 0.9 | 336 | -1.1 | 1.1 |  | -0.3 | -0.8 | 0.2 | 0.273 |
|  | 12 months | 19 | -1.4 | 0.9 | 278 | -1.0 | 1.1 |  | -0.4 | -0.9 | 0.2 | 0.163 |
|  | 18 months | 14 | -1.1 | 1.0 | 174 | -1.0 | 1.1 |  | -0.1 | -0.7 | 0.4 | 0.623 |
|  |  |  |  |  |  |  |  |  |  |  |  |  |
